# Teaching as a Catalyst: Driving Change Through Education

**DOI:** 10.1093/geroni/igaf122.397

**Published:** 2025-12-31

**Authors:** Kara Dassel

**Affiliations:** University of Utah, Salt Lake City, Utah, United States

## Abstract

When we hear the word “teaching,” many of us picture traditional classroom instruction. However, education reaches far beyond the walls of the classroom and takes on many forms across settings and disciplines. This lecture explores the diverse and dynamic nature of teaching, highlighting how meaningful educational experiences unfold within healthcare and community-based environments, through the design and implementation of interprofessional programs, through mentoring relationships with individual students, and, of course, within the classroom itself. Drawing on examples from practice, this talk will illustrate how teaching can be a collaborative, adaptive, and deeply impactful process. Whether guiding a team in a clinical setting, shaping curriculum across professional boundaries, or mentoring a student one-on-one, educators have the power to influence learning in transformative ways. Attendees will be invited to reflect on their own teaching roles and consider how education can be leveraged to create broader, lasting impact across multiple contexts.

